# Luminal lactate in acute pancreatitis - validation and relation to disease severity

**DOI:** 10.1186/1471-230X-12-40

**Published:** 2012-04-30

**Authors:** Lauri Pynnönen, Minna Minkkinen, Sari Räty, Juhani Sand, Isto Nordback, Anders Perner, Jyrki Tenhunen

**Affiliations:** 1 Departments of Critical Care Medicine, Tampere University Hospital, Teiskontie 35, Tampere, 33520, Finland; 2 Department of Gastroenterology and Alimentary tract Surgery in Tampere University Hospital, Teiskontie 35, Tampere, Finland; 3 Department of Intensive care, Copenhagen University Hospital, Rigshospitalet, Blegdamsvej 9, Copenhagen, 2100, Denmark; 4 Critical Care Medicine Research Group Tampere, Department of Critical Care Medicine, Tampere University Hospital, Teiskontie 35, Tampere, 33520, Finland; 5 Department of Surgical Sciences/Anaesthesiology and Intensive Care, University of Uppsala, Akademiska Sjukhuset ing 70, 1tr, Uppsala, 75185, Sweden

**Keywords:** Rectal luminal lactate, Acute pancreatitis, Intestinal hypoperfusion

## Abstract

**Background:**

Increased rectal luminal lactate concentration may be associated with the severity of the septic shock and high dose of vasopressors. It suggests hypoperfusion of the gut mucosa. This is potentially associated with bacterial translocation from the gut leading to local and systemic inflammation. In acute pancreatitis (AP) bacterial translocation is considered as the key event leading to infection of necrotic pancreatic tissue and high severity of illness.

**Methods:**

We used rectal luminal equilibration dialysis for the measurement of gut luminal lactate in 30 consecutive patients admitted to hospital due to acute pancreatitis to test the hypothesis that a single measurement of rectal luminal lactate predicts the severity of acute pancreatitis, the length of hospital stay, the need of intensive care and ultimately, mortality. We also tested the physiological validity of luminal lactate concentration by comparing it to luminal partial tension of oxygen. Additionally, a comparison between two different L-lactate analyzers was performed.

**Results:**

High rectal luminal lactate was associated with low mucosal partial tension of oxygen (R = 0.57, *p* = 0.005) thereby indicating the physiological validity of the method. Rectal luminal lactate at the hospital admission was not associated with the first day or the highest SOFA score, CRP level, hospital length of stay, length of stay in intensive care or mortality. In this cohort of unselected consecutive patients with acute pancreatitis we observed a tendency of increased rectal lactate in the severe cases. Low precision and high bias was observed between two lactate analyzers.

**Conclusions:**

The association between rectal luminal lactate and oxygen tension indicates that luminal lactate is a marker mucosal anaerobiosis. Comparison between two different analyzers showed poor, non-constant precision over the range of lactate concentrations. Rectal luminal lactate concentration at the time of hospital admission did not predict the severity of pancreatitis.

## Background

Inadequate blood flow and tissue ischemia or inflammation alone may induce cellular lactate release to extracellular fluid; in the intestine to luminal side of mucosa. The changes in lactate metabolism may indeed occur due to ischemia or other metabolic alterations as described by Levy and colleagues [1]. Regardless of the subcellular mechanisms increased intraluminal lactate in the rectum may be related to mucosal permeability, vasopressor dose and to the severity of illness and outcome in septic patients [2,3]. Gut luminal lactate release may occur earlier in one part of gastrointestinal (GI) tract indicating heterogeneous perfusion and metabolic changes [4].

Patients with severe acute pancreatitis develop high abdominal pressure which typically disturbs the intestinal blood flow. Systemic inflammation could initiate and maintain a disturbance in intestinal wall metabolism [3-6]. Thereafter, bacterial translocation may occur which is considered as the key event leading to infection of necrotic pancreatic tissue [7-9]. For clinicians it would be of value to detect intestinal hypoperfusion or altered metabolism early on in the clinical course of disease. Therefore, we tested the hypothesis that intestinal lactate release occurs in acute pancreatitis and that a single measurement of rectal luminal lactate predicts the severity of acute pancreatitis including highest SOFA score, length of hospital stay, need of intensive care and mortality. In an attempt to bring further credibility to this approach, we performed a physiological validation of rectal luminal lactate comparing it to luminal pO_2_. Finally, as a laboratory validation we compared two lactate-analyzers for microdialysate lactate measurement.

Multiple organ failure may be related to tissue ischemia or a broader metabolic disturbance in various tissues [3]. Until recently, tissue specific metabolic monitoring has not been possible in clinical setting: Dialysis based methods such as microdialysis [3] and equilibration dialysis [2,10-12] are options for tissue specific monitoring in general and intestinal luminal monitoring in particular. Clinical validation of the methods is inadequate. Therefore we sought to perform physiological validation and test the hypothesis that lower GI-tract hypoperfusion and metabolic changes could predict the severity of acute pancreatitis.

## Methods

This was an observational cohort study. The Local Ethical Committee approved the study protocol. Thirty consecutive patients with acute pancreatitis (regardless of etiology) were enrolled after obtaining the informed consent from the patient or a family member. One patient removed luminal equilibrium dialysis catheter before the end of the four-hour equilibration period. Data were missing from two patients. Thereby a total of 27 patients were analyzed for rectal luminal dialysate and the clinical course of the disease.

The pancreatitis diagnosis was based on typical clinical presentation, plasma amylase level exceeding three times the upper normal threshold (120 IU/L) and/or verified pancreatitis by abdominal CT-scanning or ultrasound. We determined blood leukocytes, thrombocytes, hemoglobin, C-reactive protein (CRP), creatinine (Crea), plasma bilirubin level (Bil), plasma lactate concentration (a-lact) and arterial blood gas analysis (ph, BE,pO_2_.pCO_2_). In addition, we measured Sepsis-related Organ Failure Assessment (SOFA score) at the first, third, fifth and seventh day of hospitalization. The clinically relevant complications (pseudocyst, abscess, drainage, necrotic pancreatitis, open necrosectomy) were recorded.

Equilibrium dialysis was performed on the first day of admission as a single 4-hour assessment of rectal luminal lactate concentration. Briefly, dialysis tubing (semipermeable cellulose membrane, diameter 10 mm, Sigma St Louis, MO) was attached over a 5-cm Tygon tubing (Cole-Parmer Instruments Company, Vernon Hill, IL). A three-way stopcock was used at the end to ensure airtight sampling. The bags were filled with 4 ml of 10% dextran 40 in isotonic saline (Rheomacrodex®; Meda, Sweden). The dialysis tube was placed in rectal lumen for 4 h, which is the estimated time for 100% equilibrium in vivo. When sampling the first ml was discharged as dead-space. We analyzed the sample for partial tension of O_2_ (pO_2_), L-lactate in rectal lumen (GEM 3000, Instrumentation Laboratory, MA) and CMA (600 Microdialysis Analyzer, Solna Sweden).

Comparison between two different lactate analyzers was performed: Dialysates were analyzed in parallel by using CMA600 analyzer and GEM blood gas analyzer. Comparison between the two analyzers was done by Bland-Altman plot [13].

### Primary and secondary end points

The primary endpoint was rectal luminal lactate level and Sequential Organ failure Score (SOFA). More precisely, we chose not to use Ranson Score as severity marker. Instead we used sequential organ failure assessment (SOFA) for severity depiction during the hospital stay as reported earlier [14,15].

Secondary endpoints were: 1. laboratory measurements, 2. time between debut of symptoms at the hospital admission, hydration (total volume infused intravenously prior to rectal), and the length of hospital stay 3. need of intensive care and hospital mortality.

#### Data analysis and statistics

SPSS was used for all statistical analyses. The data are presented as median (inter- quartile range, IQR). A p-value less than 0.05 was considered significant. Correlation analysis between rectal lactate (defined by GEM) and laboratory measurements, the highest SOFA, the sickness time before enrolling to the hospital, the hydration intravenously prior to rectal lactate measurement and the length of stay in the hospital was done by using Pearsons (R, p) (normal distribution) or Spearmans (*R, p) (non-normal distribution) correlation test. The comparison between two different L-lactate analyzers was performed with depicting the precision and bias with Bland-Altman plot [13]. The parallel measurement of dialysate lactate was not performed in seven cases due to inadequate sample volume.

## Results

A total of 27 patients (9 females and 18 males) with the age of 50 (40,69) years were analyzed. One of the patients needed admission to intensive care department. None of the patients died during the hospital stay. One patient was diagnosed to have colonic cancer 18 months prior to the pancreatitis diagnose and died five months after discharge for treatment of the pancreatitis. Two of the patients were discharged before the third day at hospital. Thus, no laboratory data was collected from them in the 3^rd^ 5^th^ or 7^th^ day.

The etiology of acute pancreatitis included 19 alcohol-induced, 5 gallstone-induced and 3 undetermined. The duration of symptoms was 4 (2–5) days before hospital admission. Eight of the patients developed complications; 3 pseudocysts (of which one got infected), 3 abscesses (of which all were drained and 2 were ultimately performed an open necrosectomy) and 2 necrotizing pancreatitis’ (with the other having also a thrombosis of arteria lienalis).

The total volume of intravenous fluids infused, prior to the start of equilibrium dialysis was 6800 (5300–8400) ml. The fluid therapy consisted of glucose 5%, Ringer’s acetate and 4% gelatin. At the time of the equilibrium dialysis, systemic hemodynamics were stable: mean arterial pressure (MAP) 105 (95–123) mmHg and heart rate (HR) 85 (71–98) per minute. Patients were hospitalized for duration of 7 (6–9) days.

Rectal equilibrium dialysis was performed within 1 (1–1) day after the admission and within 4 (2–5) days after the onset of symptoms. GEM lactate measurement was successful for all 27 patients.

**Figure 1:**
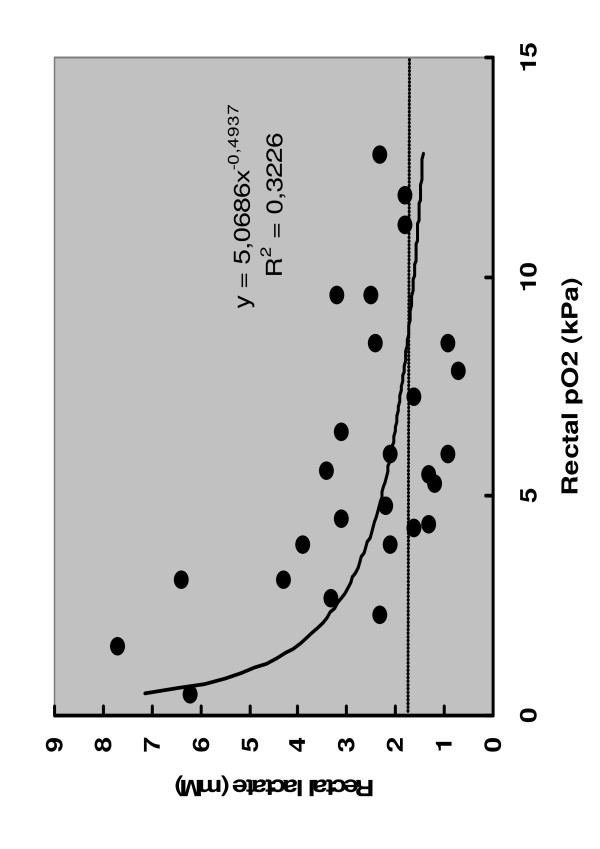
** Co-measured concentration of lactate and PO**_**2**_**in rectal dialysate from patients with acute pancreatitis (R2 = 0.3, R = -0.57,*p*** **= 0.005).**

**Figure 2:**
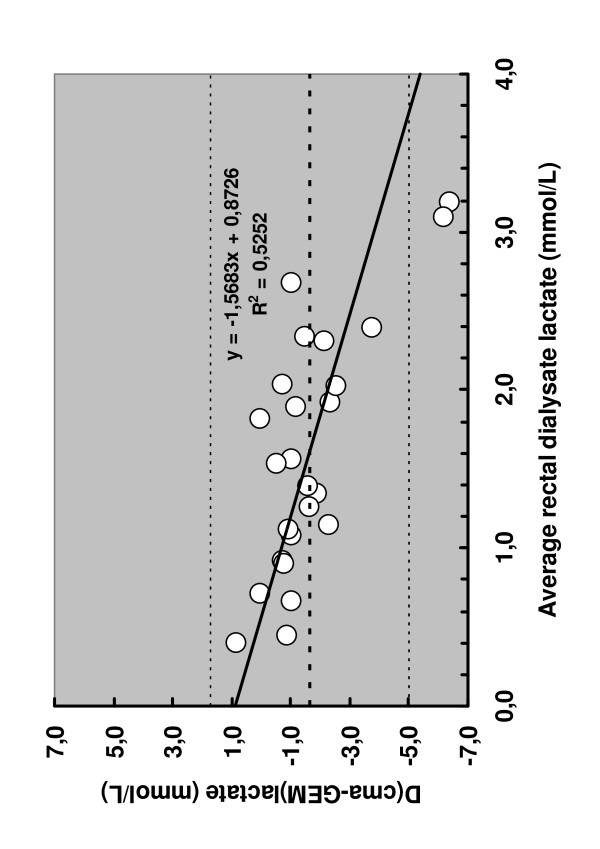
** Luminal concentrations of lactate in rectum in patients with and without clinically relevant complications from acute pancreatitis.** Data are medians (quartiles).

Dialysate lactate concentrations were correlated with dialysate PO_2_ (R = −0.57, *p* = 0.005) (Figure 1). Hydration intravenously prior to rectal lactate measurement was not correlated to rectal lactate concentration (R = −0.52, *p* = 0.79). Dialysate concentrations of lactate at the hospital admission was not correlated with duration of symptoms before hospital admission (R = −0,30, *p* = 0,13), laboratory measurements, SOFA score at the admission (Table 1), hospital length of stay (*R = −0,07, *p* = 0,7), need for intensive care or mortality. Occurrence clinically relevant complications were not associated with high rectal luminal lactate at the time of hospital admission (Figure 2). SOFA score per patient per day ranged from 0 to 6 over the length of hospital stay. Overall, SOFA score did not increase during hospitalisation medians being 2(1–3), 1(1–2) and 1(0–2) on the 3th, 5th and 7th day of hospitalisation. The highest SOFA score per patient was not correlated with the rectal lactate at the admission (R = 0.034, *p* = 0.12).

**Table 1:** Laboratory diagnostics at the hospital admission

	**1 day**		**Pearsons correlation**		
	**(n = 27)**		**R**	**p-value**	**n**
HB	133	(125–146)	-0,231	0,246	27
Hkr	0,38	(0,36–0,43)	-0,243	0,223	27
Leuc	11	(8–17)	-0,002	0,992	27
CRP	156	(70–233)	-0,16	0,424	27
Krea	63	(51–72)	*-0,31	0,115	27
Bil	21	(13–30)	*-0,14	0,486	27
trom	192	(149–215)	0,036	0,857	27
a-lakt	1,4	(1,2–2,0)	0,02	0,924	25
a-Ph	7,44	(7,42–7,47)	-0,013	0,949	27
a-Pco2	4,8	(4,5–5,2)	-0,096	0,635	27
a-Po2	10	(9–12,5)	-0,228	0,254	27
BE	1,4	(-1,5–2,8)	-0,11	0,586	27
SOFA	2	(1–3)	0,215	0,283	27

Correlations to rectal luminal lactate concentration at the admission were tested. The data are presented as median (quartiles). Herein SOFA refers to SOFA score at the time of hospital admission.

* = spearmans correlation.

**Figure 3:**
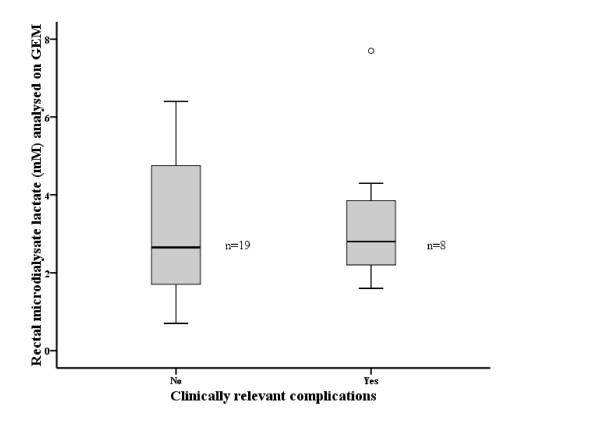
Bland-Altman analysis showed poor precision and high bias between the two methods (R2 = 0.5) with increasing bias towards higher concentrations of dialysate lactate.

Bland-Altman analysis showed poor precision and high bias between the two methods. Furthermore, the precision and Bias varied along the averaged dialysate lactate concentration (Figure 3).

## Discussion

The main finding of our study was that a single rectal lactate measurement during the first 24 h after hospital admission was not associated with the severity of illness or length of hospital stay thereby rectal luminal lactate did not predict the severity of acute pancreatitis. However, this material consisted of a cohort of unselected consecutive patients with acute pancreatitis of whom only eight had clinically relevant complication. Importantly, the physiological validation of the method indicated that increasing rectal luminal lactate was associated with low oxygen tension. Therefore, the method per se appears to depict physiological phenomena. The high bias and poor precision between two different lactate analyzers warrant further evaluation of sources of error in different analyzers.

Experimental trials suggest that the measurement of mucosal or intestinal luminal lactate by microdialysis is a valid method to investigate and depict intestinal ischemia [16,17]. Meanwhile, luminal equilibrium dialysis [11,12] is a feasible and non-invasive method for measurements rectal lactate in clinical research. It is a simple method to collect samples for measurement of markers of ischemia in rectal mucosa [11,12]. Intraluminal rectal lactate has been studied both on humans [2,10,12,17,18] and animals [3,16]. In healthy volunteers intraluminal rectal lactate is known to be 0,5 mM in average [2]. Previously, in cardiopulmonary bypass (CBP) surgery patients, rectal luminal dialysate lactate was 0,16 mM when measured prior to anaesthesia [18]. During CBP there was a 10-fold increase in the luminal lactate to 1,16 mM [18]. Similar increase to 1,6 mM was reported in severe sepsis [4] and to 4,1 mM and 4,7 mM in septic shock [2,10]. Increasing intraluminal lactate concentration in the colon positively correlated to the dose of norephinephrine used (R^2^ =0,89; *p* < 0,001) with patients under CBP [10]. In sepsis/septic shock it was first suggested that there would be an association between rectal lactate concentration and severity of the disease as well as outcome of the patients [2]. However, when studied in a larger cohort of severe sepsis patients (n = 130) there was no difference in the concentration of lactate between survivors and non-survivors [19]. In the present study the median rectal lactate concentration was 2,3 mM (1.3-6.6). This is higher than previously described normal range. On the other hand, the concentration range reported herein for the pancreatitis patients is comparable to the concentrations reported in patients under a septic shock [19]. In other words, the concentration range exceeds normal range and is comparable to concentrations in severe acute illness. In the present patient cohort we recognised eight clinically relevant complications. Only two of them were associated with high rectal luminal lactate increase (7.7 mM, 4.3 mM). Thereby, any further statistical analysis was not meaningful given the fact that as high rectal lactate concentrations as 6.2 and 6.4 mM were measured in patients without complications. Thus, occurrence of any clinically relevant complication was not associated with higher luminal lactate concentration.

Importantly, the present investigation suggests that there is a physiological rational in rectal luminal lactate measurement. As by definition, low mucosal pO_2_ was associated with presumably anaerobic release of lactate to the lumen of rectum. Alternatively, accepting the fact that gut luminal bacteria consume oxygen in addition to intestinal epithelial cells, we can only speculate that this only reflects the status of epithelial cellular aero-/anaerobiosis. Bearing this in mind it follows that a single measurement of rectal luminal lactate, and thus, rectal anaerobiosis was not associated with the need of intensive care or degree of organ failure.

The obvious limitation of the present investigation is the limited number of severe acute pancreatitis cases with only one of the patients needing intensive care. We cannot rule out the possibility of erroneously negative finding. On the other hand, eight patients developed clinically significant complication occurrence of which none was associated with high rectal luminal lactate concentration at the hospital admission. Another limitation to this study is that we collected samples describing potential anaerobiosis in only one part of GI tract. In experimental sepsis, colonic metabolic changes occur prior to changes in small intestine or stomach [4]. In more general terms, perfusion and metabolic changes are heterogeneous over the length of GI tract related to disease entity and vasoactive drugs used [20,21]. While it may seem reasonable to assume that the bacterial translocation could be induced from the large intestine, there is some evidence that in fact the small intestinal area could be of importance [8].

## Conclusions

We conclude that rectal luminal lactate concentrations were associated with luminal O_2_ tension. Contrary to our hypothesis, a single rectal luminal lactate concentration at the time of hospital admission was not predictive for the severity of pancreatitis nor did it predict the clinically relevant complications or length of hospital stay. The L-lactate analyzers used adds potentially another confounding factor to the interpretation of the results.

## Competing interests

JT was previously an advisory board member for Centricity-Clinisoft (GE) and advisory board member for dexmedetomidine in sedation for critically ill (Orion, Finland). JT is currently a member of the scientific committee for SuPAR as diagnostic tool (SuPARnostic, Denmark). JT has acted as a consultant for Eli Lilly, and travel expenses and accommodation to scientific/clinical meetings has been covered previously by Eli Lilly and Orion. Others: none.

## Authors’ contributions

LP analysed the data and wrote the manuscript, MM contributed to design, trial execution and writing, SR, JS and IN contributed to design, data analyses and writing, Ape contributed to design, data-analyses and writing, JT, contributed to design, execution, data-analyses and writing. All authors read and approved the final manuscript.

## Pre-publication history

The pre-publication history for this paper can be accessed here:

http://www.biomedcentral.com/1471-230X/12/40/prepub
